# Development and Characterization of pFluor50, a Fluorogenic-Based Kinetic Assay System for High-Throughput Inhibition Screening and Characterization of Time-Dependent Inhibition and Inhibition Type for Six Human CYPs

**DOI:** 10.3390/molecules30092032

**Published:** 2025-05-02

**Authors:** Pratik Shriwas, Andre Revnew, Sarah Roo, Alex Bender, Kevin Miller, Christopher M. Hadad, Thomas R. Lane, Sean Ekins, Craig A. McElroy

**Affiliations:** 1 Division of Medical Chemistry and Pharmacognosy, College of Pharmacy, The Ohio State University, Columbus, OH 43210, USA; 2 Department of Chemistry and Biochemistry, College of Arts and Sciences, The Ohio State University, Columbus, OH 43210, USAhadad.1@osu.edu (C.M.H.); 3 Collaborations Pharmaceuticals, Raleigh, NC 27606, USA; tom@collaborationspharma.com (T.R.L.); sean@collaborationspharma.com (S.E.)

**Keywords:** CYP450, plate reader, kinetic assay, high throughput screening, fluorescent probes, metabolism

## Abstract

Cytochrome P450s (CYPs) play an integral role in drug and xenobiotic metabolism in humans, and thus, understanding CYP inhibition and/or activation by new therapeutic candidates is an important step in the drug development process. Ideally, CYP inhibition/activation assays should be high-throughput, use commercially available components, allow for analysis of metabolism by the majority of human CYPs, and allow for kinetic analysis of inhibition type and time-dependent inhibition. Here, we developed pFluor50, a 384-well microtiter plate-based fluorogenic kinetic enzyme assay system using substrates metabolized by six human CYPs to generate fluorescent products and determined the Michaelis–Menten kinetics constants (K_M_) and product formation rates (V_max_) for each substrate–CYP pair. The pFluor50 assay was also used to elucidate inhibition type and time-dependent inhibition for some inhibitors, demonstrating its utility for characterizing the observed inhibition, even mechanism-based inhibition. The pFluor50 assay system developed in this study using commercially available components should be very useful for high-throughput screening and further characterization of potential therapeutic candidates for inhibition/activation with the most prevalent human CYPs.

## 1. Introduction

Cytochrome P450 enzymes (CYPs) are nearly ubiquitously expressed in numerous species across different domains, making them one of the most conserved superfamilies of proteins throughout life, present in archaea, bacteria, fungi, protists, animals, and even viruses [1,2]. These membrane-bound proteins are expressed throughout the human body but are most abundantly expressed in the liver, where they carry out biotransformations of both endogenous biomolecules, such as fatty acids and steroids, as well as exogenous xenobiotics [3]. The human genome comprises 57 different genes in the CYP family, including genes for 18 different families and 43 subfamilies [4,5,6,7]. However, the following six CYPs are responsible for the metabolism of more than 90% of xenobiotics in humans: CYP1A2, CYP2B6, CYP2C9, CYP2C19, CYP2D6, and CYP3A4 [8,9,10,11,12]. Additionally, CYPs can also be responsible for drug–drug interactions in the case of co-administered drugs [13,14,15]. Therefore, it is critical to understand if drug candidates are activators, inhibitors, or substrates of different CYPs [16].

The drug development process is evolving due to technological innovations but is still a time-consuming and expensive process, with drugs taking, on average, 10–12 years and 2 billion dollars of funding to get through the approval process [17]. Additionally, there is a widely noted very high attrition rate wherein only about 10% of compounds make it through the process [18]. One of the reasons for this attrition is drug metabolism and pharmacokinetics (DMPK), in which CYPs play an important role. Therefore, it is necessary to develop high-throughput methods to examine CYP–drug interactions in vitro early in the drug development pipeline [19].

Currently, the most common assays for CYP inhibition are based on chromatographic techniques such as analytical HPLC, UPLC as well as LC-MS/MS analysis. These methods are laborious and time-consuming (with some assays having run times of 5–7 min/sample or more), requiring sample preparation steps prior to injection and expensive equipment as well as skilled labor [20,21]. Additionally, kinetic data collection using these techniques requires a different sample for every timepoint, further decreasing the throughput. Another method that is often employed is fluorogenic or fluorescence-based assays. The main advantages of these systems are the adaptability to 384-well microtiter plates (making them high-throughput), no sample preparation required (there is no need to remove the proteins or other reagents from the reaction solution as fluorescence is emitted only by the product formed by the CYP metabolism of the substrate), and the ability to perform continuous real-time kinetic data collection [22,23]. However, some drugs have intrinsic fluorescence [for example, intrinsically fluorescent chemotherapeutic agents with excitation at 350 nm and emission at 450 nm] or can cause fluorescence quenching [for example, quenching by anticancer drugs] so controls must be performed to ensure that the compounds being tested do not interfere with the fluorescence of the product and having non-overlapping excitation and emission wavelengths (between the fluorescent product and the drug) is essential [24,25,26,27]. Additionally, it is possible for different substrates to have distinctive inhibition or activation profiles, so having more than one fluorogenic substrate option for a given CYP can be advantageous from this perspective as well [28].

In this study, we have developed pFluor50, a system of fluorogenic kinetic assays using substrates that form a fluorescent product through CYP metabolism with excitation wavelengths from 485 to 560 nM and emission wavelengths from 525 to 590 nM, which are non-overlapping for most intrinsically fluorescent drugs including most coumarin containing compounds [24,25,29]. Using substrates with non-overlapping wavelengths should prevent interference from the intrinsic fluorescence of drugs and also prevent quenching due to Förster resonance energy transfer. We have used the commercially available CYPexpress^TM^ system as the source of the recombinant CYPs, which is beneficial for sample preparation if further studies of metabolite production are desired and for exploring mechanism-based inhibition as low-speed centrifugation retains the CYP system for further study or removes it for metabolite production studies. Although some of the fluorogenic substrates used have been previously reported, we report the use of resorufin ethoxy ether as a novel substrate for CYP2C9 and CYP2D6. We have determined the K_M_ of the substrates with the corresponding CYPs and the V_max_ for the formation of the product. We have further validated the assays by determining the IC_50_ of known inhibitors and have demonstrated the utility of the methods for determining time-dependent inhibition and type of inhibition for some of the inhibitors.

## 2. Results

### 2.1. Calibration Curves for Different Products

The calibration curves corresponding to two of the three products (fluorescein and resorufin), along with the gain values used, can be seen in Figure S3A–F. The gain values used in the calibration curves were maintained in all other assays. All other data have been normalized to the corresponding product formation rates based on these calibration curves. Fluorescein benzyl ester (the product formed from dibenzyl fluorescein) is not commercially available, so the product formation rate could not be determined, and the V_max_ for CYP3A4 cleavage of dibenzyl fluorescein has been reported as the rate of change in relative fluorescence units (RFU) per minute. All the optimized conditions used for fluorogenic assays have been described in Table 1 and Table 2.

### 2.2. Michaelis–Menten Analysis for Determination of Kinetic Parameters of CYP–Substrate Pairs

Michaelis–Menten kinetics analysis was performed using different concentrations of the substrates, and the K_M_ and V_max_ values were determined (Table 3). The K_M_ values determined were comparable to those reported in the literature where available. Eres is a known substrate for CYP1A2, and the K_M_ value of 0.35 ± 0.1 µM is similar to the reported value of 0.62 ± 0.14, 0.26 ± 0.06, 0.56 (0.44–0.68), etc. [30,31,32,33,34]. Bzres was used as a substrate for CYP2B6, and the K_M_ value of 40.6 ± 19.6 µM was close to the previously determined value of 34.0 ± 10.4 µM [35,36]. 3OMF is a known substrate for CYP2C19, and the K_M_ value was found to be 2.3 ± 0.8 µM.

This value is similar to the previously reported K_M_ value of 1.1 ± 0.9 and 1.18 ± 0.06 µM for OMF metabolism by CYP2C19 [37,38,39]. DBF is a known substrate for CYP3A4, and the K_M_ value reported here, 1.77 ± 0.3 µM, is comparable to the literature value of 0.87 ± 0.12 and 1.37 ± 0.22 µM [40,41]. We demonstrate here that Eres is also a substrate for CYP2C9 with a K_M_ of 0.45 ± 0.06 µM. Similarly, Eres is a novel substrate for CYP2D6 with a K_M_ of 1.63 ± 0.7 µM. Thus, the fluorogenic method developed here uses Eres as a novel substrate for CYP2C9 and CYP2D6.

**Figure 1:**
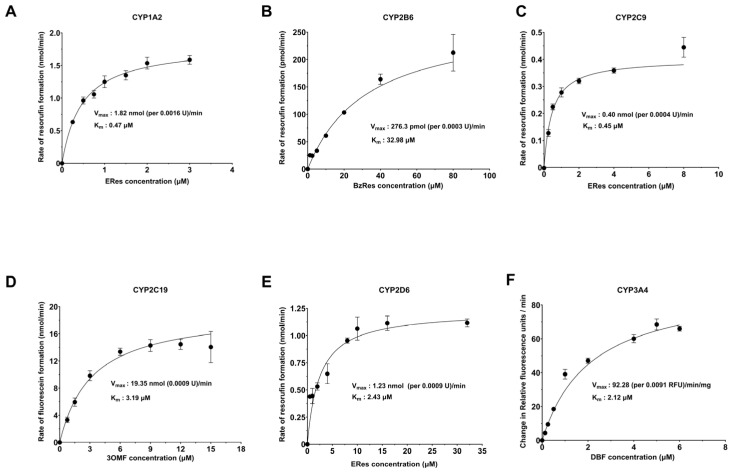
Michaelis–Menten kinetic analysis of the 6 CYP–substrate pairs. (**A**) Eres are used as substrate for 1 mg/mL CYP1A2. (**B**) Bzres are used as substrate for 1 mg/mL CYP2B6. (**C**) Eres are used as substrate for 1 mg/mL CYP2C9. (**D**) 3OMF are used as substrate for 2 mg/mL CYP2C19. (**E**) Eres are used as substrate for 1 mg/mL CYP2D6. (**F**) DBF are used as substrate for 1 mg/mL CYP3A4.

To determine the V_max_ for the formation of the products, it was assumed that the increase in fluorescence observed was only due to the formation of the final product. As discussed above, a dose-dependent fluorescence calibration curve was generated for each final product and used to calculate the concentration of product at each timepoint (with the exception of fluorescein benzyl ester). V_max_ values were then calculated from the concentration of the final product at each timepoint, yielding the rate of formation. Table 3 details the average V_max_ for each CYP–substrate pair. Overall, the Michaelis–Menten kinetics analysis demonstrated K_M_ values similar to those reported in the literature for CYP1A2, CYP2B6, CYP2C19, and CYP3A4, whereas novel probes were identified for CYP2C9 and CYP2D6. Figure 1 shows Michaelis–Menten kinetic parameters for each CYP–substrate pair [30,31,32,33,34,35,36,37,38,39,40,41].

### 2.3. Determination of the Time-Dependence of CYP Inhibition by Specific Inhibitors

**Figure 2:**
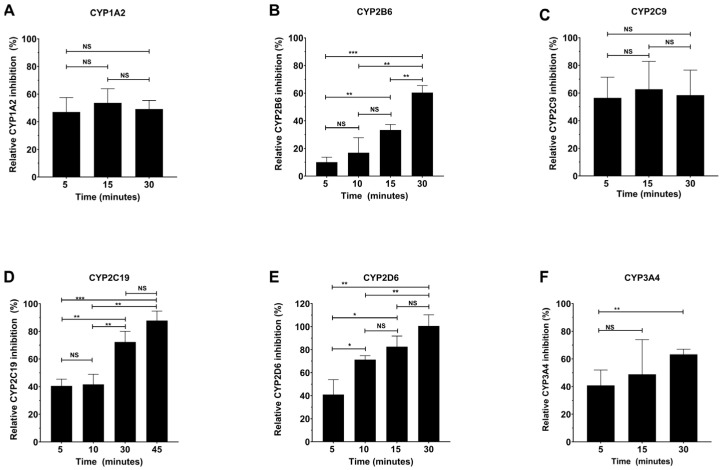
Time-dependent inhibition of CYPs using specific inhibitors. (**A**) CYP1A2 was inhibited by 0.03 µM α-naphthoflavone in a time-independent manner between 5 and 30 min. (**B**) CYP2B6 was inhibited by 30 µM sertraline in a time-dependent manner between 5 and 30 min. (**C**) CYP2C9 was inhibited by 1 µM sulfaphenazole in a time-independent manner between 5 and 30 min. (**D**) CYP2C19 was inhibited by 0.3 µM ticlopidine in a time-dependent manner between 5 and 45 min. (**E**) CYP2D6 was inhibited by 30 µM sertraline in a time-dependent manner between 5 and 30 min. (**F**) CYP3A4 was inhibited by 0.05 µM CYP3cide in a time-dependent manner between 5 and 30 min. All the experiments have been performed as three biological replicates. Each biological replicate consists of 4 technical replicates. Single-factor ANOVA was used for determination of statistical significance (NS—Not significant, * *p* ≤ 0.05, ** *p* ≤ 0.01, and *** *p* ≤ 0.001).

Interestingly, it was found that for specific CYPs, some of the selected inhibitors (some of which are FDA-approved drugs) were time-dependent, showing statistically significant differences in inhibition depending on the pre-incubation time, whereas others were independent of time (Figure 2). Therefore, the pre-incubation time between the inhibitor and the CYP was first optimized prior to the full IC_50_ determinations. Figure 2 details the time dependence for each of the CYPs with the selected inhibitors. Figure 2A shows that 30 nM α-naphthoflavone inhibits CYP1A2 in a time-independent manner, as was noted previously [42]. Therefore, a 10 min pre-incubation time was selected for convenience. It is documented that sertraline is a substrate for CYP2B6 and is converted to N-desmethylsertraline, which can also be further metabolized [43]. Figure 2B demonstrates that the inhibition of CYP2B6 with 30 µM sertraline increases with time, suggesting that the sertraline metabolites also inhibit CYP2B6, but with different inhibition constants. However, at short pre-incubation times with sertraline, the variability in the IC_50_ data was high, suggesting that the metabolism of sertraline is too fast to determine direct inhibition by sertraline without interference from the metabolites. Therefore, the sertraline pre-incubation time for the inhibition assay was optimized to 30 min (the time at which inhibition was no longer time-dependent). Nonetheless, this still led to higher variability in the CYP2B6 IC_50_. Figure 2C demonstrates that 1 µM sulfaphenazole inhibits CYP2C9 in a time-independent manner as previously established, so a 10 min pre-incubation time was selected for convenience [44]. Ticlopidine is known to be a time-dependent inhibitor as it is metabolized to an active metabolite through the action of CYP2C19 [45].

We found that the inhibition of CYP2C19 by 0.3 µM ticlopidine increased with increasing time (Figure 2D). To ensure that the inhibition being measured was due to ticlopidine rather than its metabolite, 5 min (the shortest time attainable with reagent additions and mixing) was selected as the optimal time for the IC_50_ inhibition study to determine the direct inhibition by ticlopidine. Similarly, Figure 2E demonstrates that an increase in inhibition with time was also observed for 30 µM sertraline with CYP2D6, suggesting that CYP2D6 also metabolizes sertraline and does so too quickly for measurement of direct inhibition by sertraline alone [43]. Thus, a 30 min pre-incubation time was also selected for CYP2D6 inhibition, which also led to a higher variability. It is well known that CYP3cide is a mechanism-based inhibitor of CYP3A4, and Figure 2F demonstrates that inhibition of CYP3A4 by 50 nM CYP3cide does indeed increase with time. Again, a pre-incubation time of 5 min was selected to determine direct inhibition by CYP3cide [46]. However, at longer incubation times, a shift in the IC_50_ was observed, demonstrating the utility of the method for characterizing the rate of irreversible inactivation.

### 2.4. Determination of IC_50_ for Known Inhibitors

**Figure 3:**
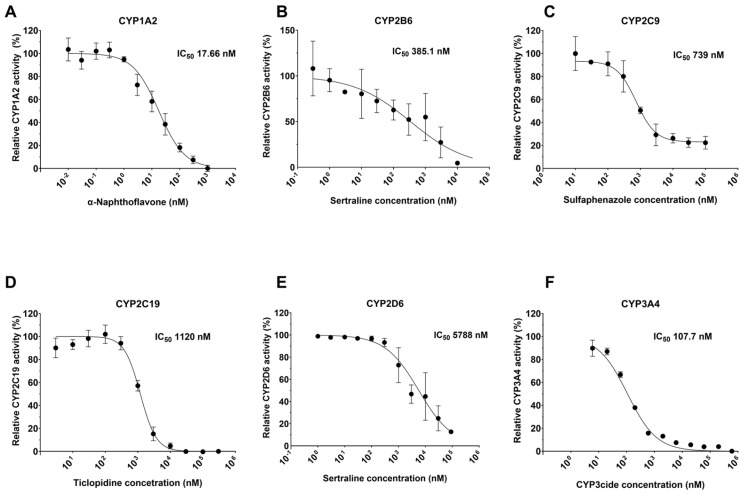
Dose-dependent inhibition of CYPs using specific inhibitors. (**A**) CYP1A2 was inhibited by α-naphthoflavone after 10 min of pre-incubation. (**B**) CYP2B6 was inhibited by sertraline after 30 min of pre-incubation. (**C**) CYP2C9 was inhibited by sulfaphenazole after 10 min of pre-incubation. (**D**) CYP2C19 was inhibited by ticlopidine after 5 min of pre-incubation (**E**) CYP2D6 was inhibited by sertraline after 30 min of pre-incubation. (**F**) CYP3A4 was inhibited by CYP3cide after 5 min of pre-incubation. IC_50_ values presented in the figure are for the biological replicate shown. All data have been collected as three biological replicates with average IC_50_ values and standard deviations presented in Table S4.

Once the pre-incubation times were optimized, full IC_50_ curves were generated for each of the six CYPs with the selected inhibitors. Figure 3 shows the IC_50_ curves as well as the calculated IC_50_ values for each of the inhibitor–CYP pairs, and Table 4 details the average and standard deviation of the IC_50_ values from three biological replicates of four technical replicates each (the data for each individual repeat is included in Table S4) and compares these values to literature data, showing good general agreement.

### 2.5. Determination of the Type of Inhibition for Select CYP–Inhibitor Pairs

**Figure 4:**
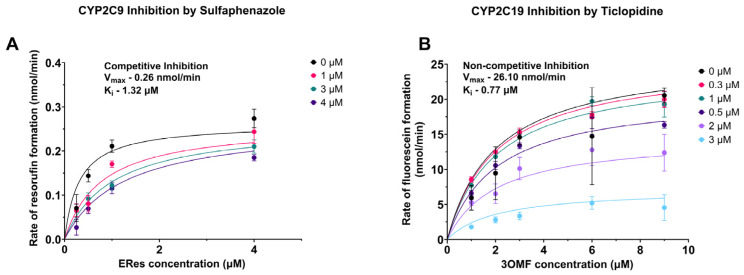
Type of Inhibition of CYP2C9 with sulfaphenazole and CYP2C19 with ticlopidine. (**A**) CYP2C9 was inhibited by sulfaphenazole in a competitive way. (**B**) CYP2C19 was inhibited by ticlopidine in a non-competitive way. K_i_ and V_max_ values presented in the figure are for the biological replicate shown. All the experiments have been performed as three biological replicates with average and standard deviation of K_i_ and V_max_ presented in Tables S5 and S6.

After determining the IC_50_ for each of the specific inhibitors, the utility of the developed method to determine the inhibition type for select CYP–inhibitor pairs was explored. The same concentrations of the substrate used in the previous Michaelis–Menten kinetics determinations were used again, along with varying concentrations of the inhibitor. Figure 4A,B show the resultant data for the inhibition of CYP2C9 by sulfaphenazole and the inhibition of CYP2C19 by ticlopidine, respectively. Sulfaphenazole inhibition of CYP2C9 yielded no change in the V_max_ as the concentration of inhibitor changed, suggesting competitive inhibition, and comparisons using Akaike’s Information Criterion selected competitive inhibition as the model that most likely produced the data. The average CYP2C9 inhibition constant, K_i_, of sulfaphenazole, was 1.43 ± 0.7 µM with an average V_max_ of 0.27 ± 0.09 nmol/min (Tables S5 and S6).

In the case of ticlopidine inhibition of CYP2C19, V_max_ clearly decreases with increasing concentrations of ticlopidine, suggesting non-competitive inhibition. Indeed, comparisons using Akaike’s Information Criterion also determine that non-competitive inhibition is the model most likely to have generated the data and fits mixed inhibition yield an alpha value close to one, also suggestive of non-competitive inhibition. The average inhibition constant K_i_ was found to be 0.70 ± 0.09 µM with an average V_max_ of 25.0 ± 3.1 nmol/min.

## 3. Discussion

In humans, CYPs play a critical role in xenobiotic metabolism and drug–drug interactions such that the determination of inhibition by and metabolism of potential therapeutics is an important step in the drug development pipeline. Therefore, a high-throughput, kinetic fluorogenic assay system with commercially available reagents that can be used for the determination of inhibition or activation of the CYPs most important for xenobiotic metabolism in humans is of critical importance [67,68,69].

Herein, we report the development of pFluor50, a set of high-throughput fluorogenic CYP assays using the commercially available CYPExpress^TM^ reagents which provide reproducible kinetic data suitable for screening compounds for CYP inhibition or activation, determining the time-dependence of that inhibition or activation, and the inhibition type. To our knowledge the fluorogenic method described herein is the only one or one of a few that has been demonstrated to determine mechanism-based or time-dependent CYP inhibition and also type of inhibition. Each CYP enzyme and master mix was optimized to include the appropriate specific reagents needed to provide linear responses within the time window selected when paired with the appropriate fluorogenic substrates (see Table 1 and Supplementary Materials). Additionally, the total incubation times and Ex:Em values were also optimized. Further, the parameters for the plate reader, such as the speed with which reads were collected, the number of measurements for every read, the total time interval between two consecutive reads, the height at which the fluorescence was detected, and the fluorescence gain values, were determined and then fixed to ensure reproducible readings every time the assay was run (Table S1) and to aid in method transfer so the assays can be easily replicated by others. All parameters were optimized to ensure the linearity of the increase in fluorescence due to product formation within the linear phase of the enzyme kinetics. Optimization was performed by empirical methods and the appropriate conditions are reported here and in the Supplementary Materials. The optimization of these plate reader parameters, along with kinetic parameters, makes the method easily reproducible and transferable, and it can be used effectively for determining CYP inhibition/activation.

Because the K_M_ can depend on many factors, including pH, temperature, and ionic strength, the methods were developed at a constant pH value of 7.4 and a constant temperature of 37 °C (physiological pH and temperature) [70,71,72]. Although K_M_ is independent of enzyme concentration, V_max_ is dependent on enzyme concentration [73,74]. In this study, we have used a commercially available proprietary enzyme mix (which contains the CYP enzymes along with the additional enzymes NAPDH oxidoreductase and G6PDH as well as MgCl_2_), wherein the supplier does not provide information on the exact quantity of each component. Therefore, we have included the amount of the CYPExpress^TM^ reagent (both mg and units) used in each case rather than the concentration of each individual component. Nonetheless, the K_M_ values obtained using the developed assays in this study were quite similar to the reported K_M_ values for the CYP–substrate pairs when values were available in the literature (Table 1), as was the case for CYP1A2, CYP2B6, CYP2C19, and CYP3A4 [30,31,32,33,34,35,36,37,38,39,40,41]. To the best of our knowledge, this is the first report on the use of Eres as a substrate for CYP2C9 and CYP2D6. In all cases where the fluorescent product was commercially available, the V_max_ was calculated as the rate of formation of the product (Table 1). However, fluorescein benzyl ester (the fluorescent product following the cleavage of dibenzyl fluorescein) is not commercially available; therefore, the V_max_ for CYP3A4 cleavage of dibenzyl fluorescein has been reported as the rate of change in relative fluorescence units (RFU) per minute.

The newly developed pFluor50 methods were further validated by determining the IC_50_ for known inhibitors of each of the six CYPs. One of the major factors that was considered when performing the inhibition studies was the selection of the solvents used to dissolve the inhibitors. The solvent choice was found to be critical because some CYPs are very sensitive to some solvents, demonstrating a significant loss in activity even in the presence of 0.3–1% final solvent concentration [75,76]. Interestingly, it was also found that the inhibition potency of specific CYPs by some of the selected inhibitors (some of which are FDA-approved drugs) was shown to be time-dependent. Therefore, the pre-incubation time between the inhibitor and the CYP was first optimized prior to IC_50_ determination. It was found that some inhibitors, such as ticlopidine, CYP3cide, and sertraline, were time-dependent while α-naphthoflavone and sulfaphenazole were time-independent inhibitors (Figure 2). Based on our analysis, the inhibition times for these inhibitors were optimized. CYP2B6 and CYP2D6 can metabolize sertraline with different potencies at low or high concentrations, and at short incubation times, the data were highly variable (likely due to metabolite formation and inhibition by metabolites), so a 30 min pre-incubation time was selected for CYP2B6 and CYP2D6 inhibition by sertraline [77,78,79,80,81,82,83]. Similarly, incubation times were optimized for ticlopidine, CYP3cide, α-naphthoflavone, and sulfaphenazole.

After optimizing the pre-incubation time, CYP inhibition assays were performed using the specific inhibitors. The IC_50_ values determined in this study (Figure 3) were largely similar to those reported in the literature [46,47,48,49,50,51,52,53,54,55,56,57,58,59,60,61,62,63,64,65,66]. It should be noted that although the pFluor50 assay methods differed from the methods used in the literature (which were traditional LC-MS/MS methods) and the substrates were also different, the determined IC_50_ values were still consistent. It is possible, if not likely, that the small differences that were observed could be due to differences in the pre-incubation times, as these were often not noted in the literature, and the most significant observed differences were in the values calculated for the inhibitors that were time dependent. Nonetheless, this demonstrates the robustness of the developed methods in comparison to the traditional methods and confirms that the developed assays are suitable for screening and characterization of drugs in the drug development pipeline. Finally, the type of inhibition was determined for CYP2C9 inhibition by sulfaphenazole and CYP2C19 inhibition by ticlopidine. This further demonstrates that the methods developed are appropriate for not only determining inhibition but also characterizing the type of inhibition.

Additionally, because the CYPExpress^TM^ reagents were selected as the source of the enzyme, further studies of metabolite production and mechanism-based inhibition would be simplified. Low-speed centrifugation could be used to remove the enzymes for the metabolite production studies, facilitating sample preparation prior to mass spectrometric determination of the metabolites produced. The same procedure could also be used to retain the enzymes allowing one to wash away the inhibitors prior to activity determination using this method to expedite the confirmation of mechanism-based inhibition.

Overall, fluorogenic assays are advantageous in several aspects compared to other more traditional CYP metabolism assays, such as LC-MS/MS- or HPLC-based methods. They tend to cost less, require less expensive and more readily available instrumentation, and require less expertise in instrument handling. Further, fluorogenic assays are easily adaptable to high-throughput methods, making them useful for screening a large number of drug candidates at a time. The pFluor50 assay system has been shown to provide comparable results to previously reported methods while providing real-time kinetic reads that enable further characterization of the observed inhibition, such as the time-dependence of the inhibition and the inhibition type (Table 1, Table 2, Table 3 and Table 4; Tables S1 and S3–S6).

## 4. Materials and Methods

### 4.1. Reagents

The following CYPexpress^TM^ CYP enzyme systems [Oxford Biomedical Research, Inc. (Rochester Hills, MI, USA); consisting of a proprietary mix of the specific CYP as well as nicotinamide adenine dinucleotide phosphate (NADPH) reductase, magnesium (Mg^2+^), NADP+ and glucose-6-phosphate dehydrogenase (G6PDH)] were purchased from Sigma Aldrich (St. Louis, MO, USA): CYP1A2 (Sigma-MTOXCE1A2), CYP2B6 (Sigma-MTOXCE2B6), CYP2C9 (Sigma-MTOXCE2C9), CYP2C19 (Sigma-MTOXCE2C19), CYP2D6 (Sigma-MTOXCE2D6), and CYP3A4 (Sigma-MTOXCE3A4). The following fluorogenic substrates were obtained from Cayman Chemical (Ann Arbor, MI, USA): 7-ethoxy-3H-phenoxazin-3-one or resorufin ethoxy ether (Eres) for CYP1A2, CYP2C9, and CYP2D6 (catalog no. 16122) and 7-(benzyloxy)-3H-phenoxazin-3-one or resorufin benzyl ether (Bzres) for CYP2B6 (catalog no. 18077). The following fluorogenic substrates were purchased from Chemodex (St. Gallen, Switzerland): 2-(6-methoxy-3-oxo-3H-xanthen-9-yl)benzoic acid or 3-O-methyl fluorescein (3OMF) for CYP2C19 (catalog no.-M0098) and benzyl 2-(6-(benzyloxy)-3-oxo-3H-xanthen-9-yl)benzoate or dibenzyl fluorescein (DBF) for CYP3A4 (catalog no. D0282). The literature precedented inhibitors were purchased from the vendors noted in parenthesis for each: α-naphthoflavone (Sigma N5757) for CYP1A2, sertraline hydrochloride (Cayman Chemical 14839) for CYP2B6 and CYP2D6, sulfaphenazole (Sigma UC166) for CYP2C9, ticlopidine hydrochloride for CYP2C19 (TCI chemicals T3110), and CYP3cide (Cayman Chemical 15019) for CYP3A4. Perkin Elmer (Waltham, MA Part number 6007270) 384-well optiplate black bottom black well plates were used for all the experiments. MgCl_2_, NADPH, NADP+, and G6P were purchased from Sigma and were HPLC grade. 18.2 MΩ MilliQ water was used to prepare 0.1 M potassium phosphate buffer (pH 7.4) with potassium phosphate monobasic (0.03042 M) and potassium phosphate dibasic (0.06958 M). This buffer was used as the incubation buffer throughout the assays. The final fluorescent metabolite formation was determined using a Biotek Synergy H1 plate reader to measure fluorescence at the noted excitation (Ex) and emission (Em) wavelengths.

### 4.2. pFluor50 Assay System Development for CYP Activity Using Fluorogenic Substrates

The concentration of each individual CYPexpress^TM^ reagent, the appropriate kinetic read time, and the appropriate read time interval were optimized to obtain linearity in the formation of the fluorescent product (steady-state kinetics) [Table 1 and Table S1]. Additionally, the concentration of each of the reagents was optimized for each CYP to generate an appropriate master mix of reagents for optimal performance and linearity during the required read times. Finally, each of the plate reader parameters (Ex, Em, gain, read height, etc.) was optimized for the individual CYP–substrate pair to minimize background fluorescence and yield the highest signal-to-noise.

In general, the protocol for determination of the Michaelis–Menten kinetic parameters (K_M_ and V_max_) involved addition of the appropriate master mix to phosphate buffer followed by addition of the appropriate amount of enzyme to the mix. The master mix was determined for each specific CPY450 based on empirical methods involving each reagent to obtain a linear range of enzyme activity. A total of 30 µL of this enzyme mix was then added to each well of the 384-well plate. Next, initial background readings were taken for 5 min, after which the appropriate amount of substrate was dispensed into each well using the onboard reagent dispenser of the plate reader. All plates contained one blank well without enzyme (only master mix) for each substrate concentration. Figure S1 shows the layout of the well plate for determining the K_M_ for CYP3A4 as an example. Figure S2 shows the structures of substrates and final products. Table 1 provides the formulation of the master mix for each CYP, the kinetic read time with substrate, the read time interval, the concentration range of the substrate used for the Michaelis–Menten kinetics, the Ex and Em wavelengths, and the concentration of substrate for inhibition/activation screening. Table S1 details the specific parameters used for the plate reader for each CYP–substrate pair.

### 4.3. Michaelis–Menten Kinetics for Each CYP–Substrate Pair

For determining the Michaelis–Menten kinetic parameters (K_M_ and V_max_) for each CYP–substrate pair, kinetic studies were performed in which a range of different concentrations of the substrate were used [84]. Table 1 details the formulations for the master mix, CYPexpress^TM^ concentrations, and concentration ranges of each specific substrate. For each CYP–substrate pair, plate reader parameters were set up as described in Table S1. The protocol used for Michaelis–Menten kinetics was similar for all the CYPs. Briefly, the working solution was prepared for each substrate and then primed into one of the dispensers on the plate reader. The other dispenser was primed with the buffer solution. 1.5 mL of phosphate buffer solution (pH 7.4) was used to prepare the master mix with the appropriate concentration of each reagent as specified in Table 1 (NADPH, NADP+, G6P, G6PDH, and/or MgCl_2_). The amount of CYPexpress reagent in units/(mg/mL) was determined for each enzyme as new lots may have slightly different activities, and the activity of the reagent may change over time. The appropriate mass of each CYPexpress^TM^ reagent needed to obtain the required units was measured using an analytical balance and was transferred to the master mix solution. This solution was mixed, and 30 µL of the solution was added to each well using a multichannel pipette. In each case, a blank control well was also included, as described in Figure S1. The plate was then transferred to the plate reader and readings were taken at the time intervals, for the appropriate length of time, and at the appropriate wavelengths as specified in Table 1.

### 4.4. Determination of IC_50_ Values for Each CYP-Specific Inhibitor

The CYP inhibition studies used the concentrations of substrate selected based on the K_M_ values obtained in the kinetics studies above and are detailed in Table 2. The conditions of the inhibition studies were similar to those used in the kinetics experiments for the master mix formulation, enzyme amount, and assay time, but with different concentrations of inhibitors added (Table 2). It is noted that the control inhibitors selected had different mechanisms of action (some have a time dependence while others do not), so the incubation time for the enzyme–inhibitor interaction was also optimized (Table 2), and inhibition was allowed to occur for the appropriate time prior to addition of the substrate. The solvent used for each inhibitor is also noted in Table 2, as well as the final concentration of the solvent, which was maintained for each concentration tested. The same amount of solvent was added to the positive and negative control to ensure that any solvent effects were appropriately normalized.

### 4.5. Determination of Product Formation Rate

Calibration curves were constructed using the determined plate reader settings (Figure S3) and concentrations of the products formed to allow for the calculation of product formation rates based on the observed fluorescence. For these calculations, we presumed that the total increase in observed fluorescence over time was due only to product formation. Calibration curves were generated with 6 different concentrations of the products (fluorescein and resorufin), which spanned the relative fluorescence units (RFU) observed during the kinetic experiments. This allowed for the calculation of product concentrations from the observed RFU, enabling the calculation of the product formation rates. Unfortunately, fluorescein benzyl ester (the product formed from the metabolism of dibenzyl fluorescein) is not commercially available. Therefore, the exact rate of formation could not be determined, and the V_max_ for CYP3A4 cleavage of dibenzyl fluorescein has been reported as the rate of change in relative fluorescence units (RFUs) per minute.

### 4.6. Data Analysis

Slopes were calculated from the collected data by determining the increase in RFUs with respect to time. For the determination of kinetic parameters (K_M_ and V_max_), the slopes were then used as the input for non-linear regression fitting using a Michaelis–Menten kinetics model for a single enzyme. For determining the IC_50_ of the inhibitors, the slopes were used as the input for non-linear regression fitting using the following equation

(1) Y=Bottom+(Top−Bottom)/(1+10^(LogIC50−X))

where X = the log of the substrate concentration, Y is the change in fluorescence intensity (slope), Top is the slope at full activity (uninhibited), and Bottom is the slope when fully inhibited [85].

All data analysis was performed using Prism 9.0 software (Graphpad Software Inc., Boston, MA, USA). Experiments were performed with three biological replicates of four technical replicates per experimental condition, and the values reported are the average of these experiments. Single-factor analysis of variance (ANOVA) was used to determine statistical significance and established with *p*-value (* *p* ≤ 0.05, ** *p* ≤ 0.01, and *** *p* ≤ 0.001).

## 5. Conclusions

Here, pFluor50, a novel fluorogenic assay system is described that has been developed to determine the inhibition of six human CYPs using the commercially available CYPExpress^TM^ system. In this assay system, we have used Eres as a novel fluorogenic substrate for CYP2C9 and CYP2D6. The pFluor50 system described can be used to provide data critical to the early stages of the drug development process and is among the few fluorogenic methods that have been shown to determine the time dependence of inhibition, which can provide insight into the mechanism of action of the inhibitors, as well as determine the type of inhibition. The approach outlined herein can be used to provide data in a high-throughput manner (faster than HPLC, etc.) on six different human CYPs (CYP1A2, CYP2B6, CYP2C9, CYP2C19, CYP2D6, and CYP3A4) that are responsible for the metabolism of >90% of xenobiotics. In the future, we would also like to develop similar methods with CYP2C8 that will comply with ICH M12 guidelines along with its inhibitor montelukast [86,87]. Known CYP-specific inhibitors were used to validate the methods and demonstrate the utility of the methods for determining the type of inhibition. The developed methods can, therefore, be used for rapid screening of drug candidates for inhibition or activation of CYP metabolism and then further characterize any observed changes.

## Figures and Tables

**Table 1:** CYP–substrate assay conditions for K_M_ determination.

CYPexpress (Amount)	CYPexpress Activity [Units/(mg/mL)] ^1^	Substrate	Ex:Em (nm)	Read Time (min)	Read Time Interval (sec)	Master Mix	Substrate Concentration Range (µM)
CYP1A2 (1 mg/mL)	CYP1A2 (0.0016 U)	Eres	560:590	10	60	NADPH-0.52 mM MgCl_2_-2.64 mM	0.25–3
CYP2B6 (1 mg/mL)	CYP2B6 (0.0003 U)	Bzres	535:590	60	90	NADPH-0.33 mM MgCl_2_-3.3 mM NADP+-1.3 mM G6P-3.3 mM G6PDH-0.4 unit/mL	1.25–80
CYP2C9 (1 mg/mL)	CYP2C9 (0.0004 U)	Eres	560:590	40	120	NADPH-0.65 mM MgCl_2_-3.3 mM	0.25–8
CYP2C19 (2 mg/mL)	CYP2C19 (0.0009 U)	3OMF	485:525	20	30	NADPH-0.33 mM MgCl_2_-3.3 mM NADP+-1.3 mM G6P-3.3 mM G6PDH-0.4 unit/mL	1–12
CYP2D6 (1 mg/mL)	CYP2D6 (0.0009 U)	Eres	560:590	75	90	NADPH-2.6 mM MgCl_2_-3.3 mM	1–32
CYP3A4 (1 mg/mL)	CYP3A4 (91.2 RFU/min/ (mg/mL) ^2^)	DBF	485:535	20	30	NADPH-0.33 mM MgCl_2_-3.3 mM NADP+-1.3 mM G6P-3.3 mM G6PDH-0.4 unit/mL	0.25–6

^1^ units/(mg/mL) calculations represents number of units of CYPExpress^TM^ reagent used at V_max_ of for each experiment (conversion 1 unit/min product formation or RFU/min for CYP3A4). ^2^ Relative fluorescence units (RFU)/min was used for CYP3A4 since the product was not commercially available for calibration to determine the actual product formation rate.

**Table 2:** CYP–substrate assay conditions for IC_50_ determination.

CYPexpress	Substrate (Concentration)	Inhibitor	Solvent (Final Concentration)	Pre-Incubation Time (min)	Inhibitor Concentrations (µM)
CYP1A2 (1 mg/mL)	Eres (2 µM)	α-Naphthoflavone	DMSO (1%)	10	0.00001–1
CYP2B6 (1 mg/mL)	Bzres (10 µM)	Sertraline	Methanol (0.5%)	30	0.0003–30
CYP2C9 (1 mg/mL)	Eres (2 µM)	Sulfaphenazole	DMSO (1%)	10	0.003–300
CYP2C19 (2 mg/mL)	3OMF (2 µM)	Ticlopidine	Acetonitrile (1.67%)	5	0.0003–300
CYP2D6 (1 mg/mL)	Eres (2 µM)	Sertraline	Methanol (0.5%)	30	0.001–100
CYP3A4 (1 mg/mL)	DBF (4 µM)	CYP3cide	DMSO (0.5%)	5	0.001–100

**Table 3:** Michaelis–Menten kinetic parameters for different CYP–substrate.

CYP (Substrate)	Average V_max_ (nmol/min)	Average K_M_ (µM)	Literature K_M_ (µM)	Ref
CYP1A2 (Eres)	1.61 ± 0.2	0.35 ± 0.1	0.62 ± 0.14 1.72 ± 0.24 0.56 ± 0.13 0.22 0.26 ± 0.06	[30,31,32,33,34]
CYP2B6 (Bzres)	0.33 ± 0.1	40.61 ± 19.6	34.0 ± 10.4 1.3 ± 0.1 (human Bl cell)	[35,36]
CYP2C9 (Eres)	0.43 ± 0.05	0.45 ± 0.0	NA	NA
CYP2C19 (3OMF)	18.2 ± 2.0	2.31 ± 0.8	1.1 ± 0.9 1.18 ± 0.06 2.32	[37,38,39]
CYP2D6 (Eres)	0.93 ± 0.2	1.63 ± 0.7	NA	NA
CYP3A4 (DBF)	91.20 ± 13.9 (RFU/min)	1.77 ± 0.3	0.87 ± 0.12 1.37 ± 0.22	[40,41]

**Table 4:** Average IC50 values for each CYP–inhibitor pair.

CYP (Inhibitor)	Average IC50 (nM)	Literature IC50 nM [Reference]	Literature Substrate/Method
CYP1A2 (*α-Naphthoflavone*)	12.2 ± 4.7	6.1 ± 0.8 [47] 8.8 ± 2.5 [48] 6 [49] 0.15 ± 0.04 [50] 9.1 [51]	Phenacetin/LC-MS/MS Fluoregenic (Eres) Fluoregenic (Eres) Acetominaphen/LC-MS/MS Phenacetin/LC-MS/MS
CYP2B6 (*Sertraline*)	751.9 ± 557.9	200 ± 24 [52] 3200 ± 900 HLM [53] 1430 (1070–1900) [54]	Bupropion/LC-MS/MS Bupropion/LC-MS/MS Bupropion/LC-MS/MS
CYP2C9 (*Sulfaphenazole*)	724.5 ± 89.8	600 [55] 480 ± 50 [56] 400 [57] 700 ± 80 [58]	Teinilic acid/LC-MS/MS Phenytoin/HPLC Diclofenac/LC-MS/MS Diclofenac/LC-MS/MS
CYP2C19 (*Ticlopidine*)	781.9 ± 413.1	370 ± 70 [59] 203 ± 124 [60] K_i_ 1200 ± 500 [61] 640 [62] 141 [63]	(S)-Mephenytoin/LC-MS/MS (S)-Mephenytoin/LC-MS/MS (S)-Mephenytoin/LC-MS/MS (S)-Mephenytoin/LC-MS/MS (S)-Mephenytoin/LC-MS/MS
CYP2D6 (*Sertraline*)	4417 ± 1292	4350 ± 500 [64] 2000 [65]	Primaquine/LC-MS/MS Bufuralol/LC-MS/MS
CYP3A4 (*CYP3cide*)	126.1 ± 66.2	300 ± 20 [46] 690 ± 22 [46] 273 (160–443) [66] 119 (78–177) [66]	Midazolam/LC-MS/MS Testosterone/LC-MS/MS DBF/fluoregenic Midazolam/LC-MS/MS

## Data Availability

Data are contained within the article.

## Supplementary Materials

The following supporting information can be downloaded at: https://www.mdpi.com/article/10.3390/molecules30092032/s1, Table S1: Synergy H1 Biotek plate parameters used during fluorogenic CYP450 assays; Table S2: Michaelis–Menten kinetic parameters for different CYP–substrate pairs; Table S3: Vmax (rate of product formation) for different CYP–substrate pairs; Table S4: Average IC50 values for each CYP–inhibitor pair (three biological replicates); Table S5: Average Ki for type of inhibition analysis (three biological replicates); Table S6: Average Vmax for type of inhibition analysis (three biological replicates); Figure S1: Plate layout for Michaelis–Menten analysis using KM substrate; Figure S2: CYP–substrate pairs used in fluorogenic assay development; Figure S3: Calibration curves for Resorufin and Fluorescein.

## Abbreviations

The following abbreviations are used in this manuscript:

CYP450	Cytochrome 450 enzymes
CYPs	Cytochrome P450
CYP1A2	Cytochrome P450-1A2
CYP2B6	Cytochrome P450-2B6
CYP2C9	Cytochrome P450-2C9
CYP2C19	Cytochrome P450-2C19
CYP2D6	Cytochrome P450-2D6
CYP3A4	Cytochrome P450-3A4
Eres	Resorufin ethoxy ether
Bzres	Resorufin benzyl ether
3OMF	3-O-methyl fluorescein
DBF	Dibenzyl fluorescein
