# Supplementary material for: Development and Characterization of pFluor50, a Fluorogenic-Based Kinetic Assay System for High-Throughput Inhibition Screening and Characterization of Time-Dependent Inhibition and Inhibition Type for Six Human CYPs
Source: Molecules. 2025 May 2;30(9):2032. doi: 10.3390/molecules30092032 (PMC12074421; doi:10.3390/molecules30092032)
Supplement: Supplementary file 1 [file molecules-30-02032-s001.zip › molecules-3599556-supplementary.pdf]

Development and Characterization of pFluor50, a Fluorogenic-Based Kinetic Assay System for High-Throughput Inhibition Screening and Characterization of Time-Dependent Inhibition and Inhibition Type for Six Human CYPs

Pratik Shriwas [1], Andre Revnew [1], Sarah Roo [1], Alex Bender [1], Kevin Miller [2], Christopher M. Hadad [2], Thomas R. Lane [3], Sean Ekins [3] and Craig A. McElroy [1,*]

[1] College of Pharmacy, Division of Medical Chemistry and Pharmacognosy, The Ohio State University, Columbus, OH 43210, USA
[2] Department of Chemistry and Biochemistry, College of Arts and Sciences, The Ohio State University, Columbus, OH 43210, USA; hadad.1@osu.edu (C.M.H.)
[3] Collaborations Pharmaceuticals, Raleigh, NC 27606, USA; tom@collaborationspharma.com (T.R.L.); sean@collaborationspharma.com (S.E.)
* Correspondence: mcelroy.31@osu.edu

Supplementary Material

1 Supplementary Figures and Tables

1.1 Supplementary Tables

Supplementary Table S1. Synergy H1 Biotek plate parameters used during fluorogenic CYP450

CYP-substrate	Speed	Delay (msec)	Measurements per read	Time interval (min)	Height (mM)	Gain	Reading time for linear region (min)
CYP1A2	Normal	100	45/read	1.5	9.75	100	0-10
CYP2B6	Normal	100	75/read	1.5	9.75	130	0-60
CYP2C9	Normal	100	100/read	2	9.75	100	0-40
CYP2C19	Normal	100	20/read	0.5	9.5	68	0-20
CYP2D6	Normal	100	75/read	1.5	9.75	100	0-75
CYP3A4	Normal	100	17/read	1	9.5	100	15-35

Supplementary Table S2. Michaelis Menten kinetic parameters for different CYP-substrate pairs

CYP-Substrate	K_M – repeat 1 (95% CI)	K_M – repeat 2 (95% CI)	K_M – repeat 3 (95% CI)	Average K_M
CYP1A2-Eres	0.33 μM (0.16-0.69)	0.225 μM (0.13-0.46)	0.47 μM (0.41-0.54)	0.35 ± 0.1 μM
CYP2B6-Bzres	32.98 μM (13.95-123.8)	25.91 μM (16.90.-41.78)	62.94 μM (40.64-104.8)	40.61 ± 19.6 μM
CYP2C9-Eres	0.46 μM (0.29-0.69)	0.52 μM (0.32-0.80)	0.39 μM (0.14-0.79)	0.45 ± 0.06 μM
CYP2C19-3OMF	3.19 μM (2.32-4.45)	1.42 μM (1.11-1.85)	2.30 μM (1.87-2.87)	2.31 ± 0.8 μM
CYP2D6-Eres	2.43 μM (1.80-3.23)	0.89 μM (0.51-1.39)	1.57 μM (0.79.-2.76)	1.63 ± 0.7 μM
CYP3A4-DBF	2.12 μM (1.70-2.67)	1.56 μM (1.08-2.20)	1.61 μM (1.27-2/04)	1.77 ± 0.3 μM

Supplementary Table S3. V_{max} (rate of product formation) for different CYP-substrate pairs

CYP-Substrate	V_{max} – repeat 1 (95% CI)	V_{max} – repeat 2 (95% CI)	V_{max} – repeat 3 (95% CI)	Average V_{max}
CYP1A2-Eres	1.64 nmol/min (1.36-2.08)	1.38 nmol/min (1.09-1.74)	1.82 nmol/min (1.72-1.93) nm	1.61 ± 0.2 nmol/min
CYP2B6-Bzres	276.3 pmol/min	264.2 pmol/min	462.4 pmol/min	334.3±111.1

	(168.4-751.3)	(195.5-371.5)	(349.6-651.6)	pmol/min
CYP2C9-Eres	0.40 nmol/min (0.36-0.45)	0.39 nmol/min (0.33-0.46)	0.49 nmol/min (0.42-0.59)	0.43±0.05 nmol/min
CYP2C19-3OMF	19.35 nmol/min (17.12-22.18) μM	15.86 nmol/min (14.13-17.98) μM	19.37 nmol/min (17.07-22.14) μM	18.2±2.0 nmol/min
CYP2D6-Eres	1.22 nmol/min (114-1.32)	0.72 nmol/min (0.60-0.85)	1.57 μM nmol/min (0.65-1.12)	0.93±0.2 nmol/min
CYP3A4-DBF	92.28 (RFU/min) (80.73-105.80)	104.6 (RFU/min) (93.92-118.0)	76.71 (RFU/min) (63.7-91.74)	91.20±13.9 (RFU/min)

Supplementary Table S4. Average IC_{50} values for each CYP-inhibitor pair (three biological replicates)

CYP-inhibitor	IC_{50} (nM)– repeat 1 (95% CI) nM	IC_{50} (nM)– repeat 2 (95% CI) nM	IC_{50} (nM)– repeat 3 (95% CI) nM	Average IC_{50} (nM)
CYP1A2- α-Naphthaflavone	17.66 (13.64-22.82)	9.27 (4.28-15.35)	9.65 (7.77-11.85)	12.2 ± 4.7
CYP2B6-Sertraline	385.1 (230.8-726.3)	1394 (1017-1994)	476.7 (205.1-1413)	751.9 ± 557.9
CYP2C9-Sulfaphenazole	738.6 (481.5-868.0)	806.5 (673.6-1287)	628.5 (257.1-823.6)	724.5 ± 89.8
CYP2C19-Ticlopidine	1193 (1015-1407)	367.4 (253.1-550.1)	806.9 (281.2-4428)	781.9 ± 413.1
CYP2D6-Sertraline	5788 (2726-12412)	3222 (2011-4935)	4241 (2363-8220)	4417 ± 1292
CYP3A4-CYP3cide	107.7 (68.72-147.6)	199.8 (149.3-272.0)	70.84 (34.42-176.3)	126.1 ± 66.2

Supplementary Table S5. Average K_i for type of inhibition analysis (three biological replicates)

CYP-inhibitor	K_i – repeat 1 (95% CI)	K_i – repeat 2 (95% CI)	K_i – repeat 3 (95% CI)	Average K_i (μM)
CYP2C9-Sulfaphenazole	1.32 μM (0.62-2.85)	0.73 μM (0.32-1.28)	2.25 μM (1.39-3.92)	1.43 ± 0.7
CYP2C19-Ticlopidine	0.77 μM (0.63-0.93)	0.75 μM (0.61-0.93)	0.59 μM (0.44-0.77)	0.70 ± 0.09

Supplementary Table S6. Average V_{max} for type of inhibition analysis (three biological replicates)

CYP-inhibitor	V_{max} – repeat 1 (95% CI)	V_{max} – repeat 2 (95% CI)	V_{max} – repeat 3 (95% CI)	Average V_{max}

CYP2C9-Sulfaphenazole	0.26 nmol/min (0.22-0.30)	0.37 nmol/min (0.34-0.40)	0.19 nmol/min (0.14-0.26)	0.27 ± 0.09 nmol/min
CYP2C19-Ticlopidine	26.10 nmol/min (24.44-27.93)	27.46 nmol/min (22.18-35.70)	21.44 nmol/min (15.07-33.88)	25.00 ± 3.1 nmol/min

1.2 Supplementary Figures

Supplementary Figure S1. Plate layout for Michaelis-Menten analysis using K_M

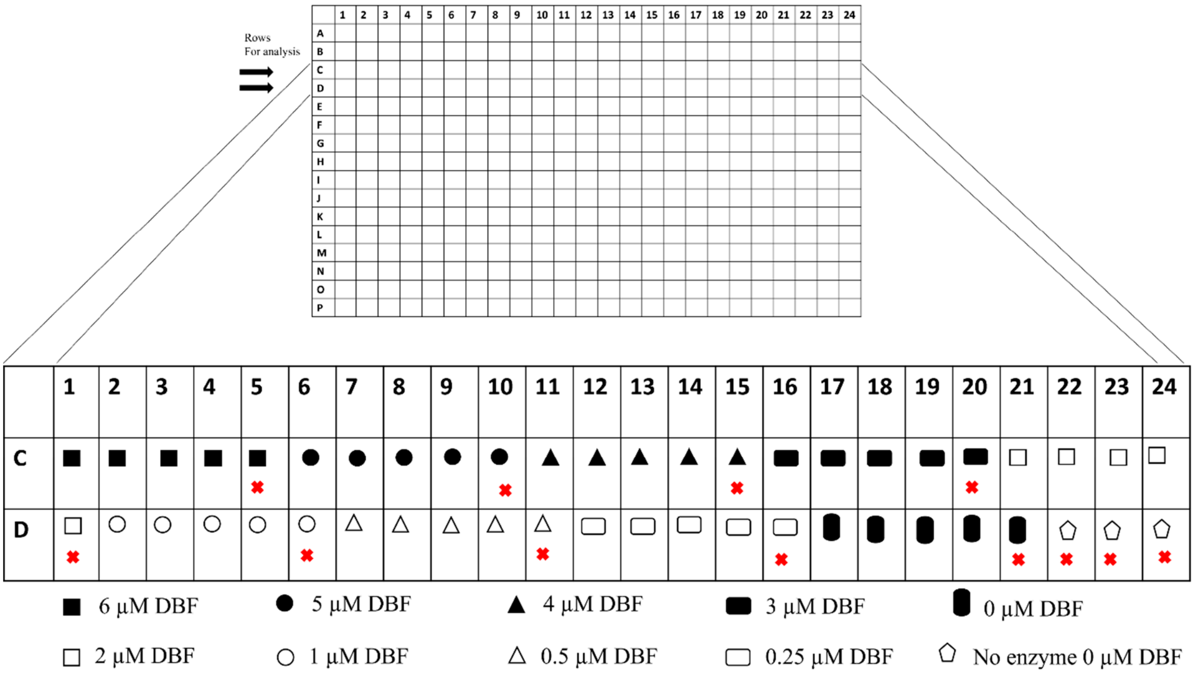

384 blackwell opti-plate layout for performing Michaelis-Menten analysis using CYP3A4 enzyme and DBF substrate. Different concentrations of substrate DBF were used in 4 replicates and a blank was used without the enzyme (✖) but with the substrate for each concentration of DBF. (⬠) Another control was used in which no enzyme as well as no inhibitor was used. This was further used to normalize all the readings.

Supplementary Figure S2. CYP-substrate pairs used in fluorogenic assay development

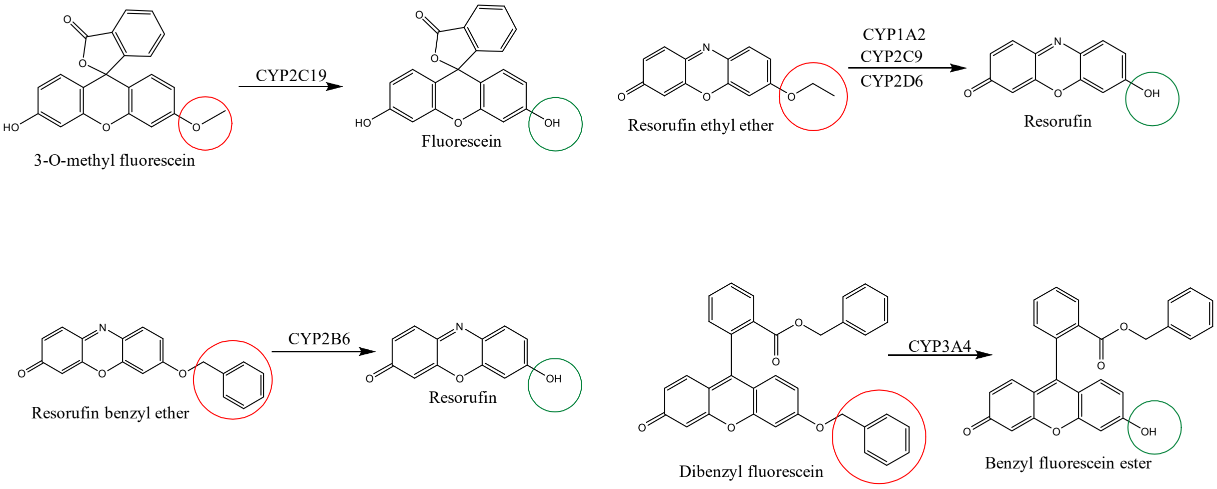

Structures of substrates and their final products used in fluorogenic assay development. The groups circled in red are metabolized to the ones in green by the specific CYP450 enzymes.

Supplementary Figure S3. Calibration curves for Resorufin and Fluorescein

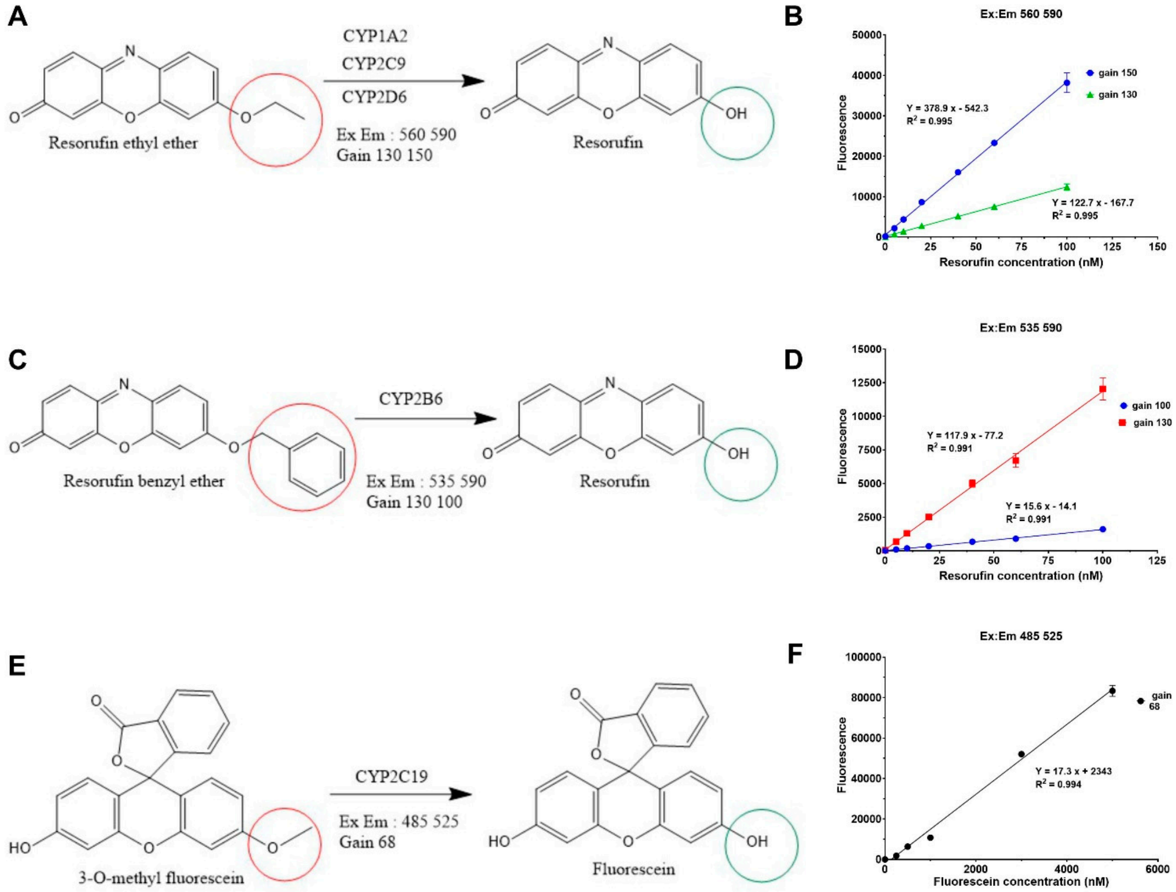

(A) Ex:Em (with gain values 150 and 130) values for formation of resorufin from resorufin ethyl ester by CYP1A2, CYP2D6 and CYP2C9. (B) Fluorescence values for resorufin (0 – 100 nM) at Ex:Em 560:590 (gain 150 and 130). (C) Ex:Em (with gain values 100 and 130) values for formation of formation of resorufin from resorufin benzyl ester by CYP2B6. (D) Fluorescence calibration curve for resorufin (0 – 100 nM) at Ex:Em 535:590 (gain 100 and 130). (E) Ex:Em (with gain value 68) values for formation of fluorescein from 3-O-Methyl fluorescein by CYP2C19. (F) Fluorescence calibration curve for fluorescein (0 – 5000 nM) at Ex:Em 485:520 (gain 68).
